# Domes and semi-capsules as model systems for infrared microspectroscopy of biological cells

**DOI:** 10.1038/s41598-023-30130-z

**Published:** 2023-02-23

**Authors:** Johanne Heitmann Solheim, Maren Anna Brandsrud, Beibei Kong, Akos Banyasz, Ferenc Borondics, Guillaume Micouin, Stine Lossius, Josep Sulé-Suso, Reinhold Blümel, Achim Kohler

**Affiliations:** 1 Faculty of Science and Technology, Norwegian University of Life Sciences, 1430 Aas, Norway; 2 Laboratoire de Chimie de l’ENS de Lyon, French National Centre for Scientific Research (CNRS), 69364 Lyon, France; 3 Synchrotron SOLEIL, L’Orme des Merisiers, Saint-Aubin-BP48, Gif-sur-Yvette CEDEX France; 4 School of Pharmacy and Bioengineering, Cancer Centre, University Hospitals of North Midlands, Keele University, Stoke on Trent, ST4 6QG UK; 5 Department of Physics, Wesleyan University, Middletown, CT USA

**Keywords:** Biological physics, Biophotonics, Micro-optics, Infrared spectroscopy

## Abstract

It is well known that infrared microscopy of micrometer sized samples suffers from strong scattering distortions, attributed to Mie scattering. The state-of-the-art preprocessing technique for modelling and removing Mie scattering features from infrared absorbance spectra of biological samples is built on a meta model for perfect spheres. However, non-spherical cell shapes are the norm rather than the exception, and it is therefore highly relevant to evaluate the validity of this preprocessing technique for deformed spherical systems. Addressing these cases, we investigate both numerically and experimentally the absorbance spectra of 3D-printed individual domes, rows of up to five domes, two domes with varying distance, and semi-capsules of varying lengths as model systems of deformed individual cells and small cell clusters. We find that coupling effects between individual domes are small, corroborating previous related literature results for spheres. Further, we point out and illustrate with examples that, while optical reciprocity guarantees the same extinction efficiency for top vs. bottom illumination, a scatterer’s internal field may be vastly different in these two situations. Finally, we demonstrate that the ME-EMSC model for preprocessing infrared spectra from spherical biological systems is valid also for deformed spherical systems.

## Introduction

To date, several powerful techniques are available for conducting vibrational spectroscopy of biological and inanimate samples in the mid-infrared spectral range. Among them are, for instance, Raman spectroscopy^1^, neutron spectroscopy^2^, photoacoustic spectroscopy^3–6^, and electron energy loss spectroscopy^7^. However, by far the most widely used analytical technique for biochemical characterization of different samples is Fourier-transform infrared spectroscopy (FTIR)^8–11^. Thus, further increasing the power of this technique and extension of its applicability is the focus of this paper. While acquisition, correction, and interpretation of biological FTIR thin-film spectra are based on established techniques, much work remains to be done in the area of FTIR spectroscopy of individual biological cells and small aggregates of cells. It is well known that biological cells that are of approximately the same size as the wavelength of infrared radiation, are highly effective scatterers. Strong scattering signatures observed in infrared spectroscopy of human cells were attributed to Mie-type scattering by Mohlenhoff et al. in 2005^12^. Since then, spherical model systems have frequently been used to model and remove scattering signatures from biological systems^13–16^. The two main contributions from Mie scattering are wiggles and ripples^17^. Wiggles are long-range oscillations caused by an interference effect^17^. They determine the average behavior of infrared extinction spectra. Ripples are sharp peaks superimposed on the wiggles. They arise due to shape resonances, i.e., standing waves inside the sample^18^.

However, it is not well understood whether spherical model systems are suitable for describing biological cells in infrared spectroscopic measurements. Most biological cells are not expected to be perfectly spherical, and different shapes are commonly observed in nature. Examples include bacteria, yeasts and algae, which exhibit a wide range of shapes, from spherical shapes to spheroids and elongated capsule shapes. In addition, when a cell is deposited onto an infrared microscope slide, it may attach to the slide, resulting in a shape deformation. In contrast, structurally stable samples, for example pollen grains, are expected to keep their spherical shape when deposited onto a slide. This can be seen with optical microscopy of pollen grains. In infrared absorbance measurements, pollen grains display close to perfect Mie-scattering signatures, with both wiggles and ripples^19^. Ripples are sensitive to the shape of the scatterer, and it has been shown that shape deformations can suppress or even eliminate ripples in infrared spectra^20^. Therefore, in addition to direct observation with optical microscopy, the presence of ripples is a further indication that pollen grains retain their spherical shape.

When biological cells are investigated using infrared spectroscopy, they often occur in an assembly. Typical sample configurations in infrared spectroscopy are eukaryotic cells in tissue, or multiple single cells deposited onto a substrate. It is therefore important to know whether neighboring cells experience coupling effects that may affect the scattering signatures.

The aims of this study are (1) to describe the scattering signatures in dome-shaped (hemispherical) systems and elongated, dome-shaped systems (semi-capsules), (2) to understand if coupling effects between adjacent particles (e.g., domes) occur, (3) to compare qualitatively numerically simulated results with spectra obtained via Fourier-transform infrared spectroscopy (FTIR), using both a synchrotron source (SR-FTIR) and a globar source with a focal plane array (FPA) imaging detector, (4) to investigate the effect of the reversal of illumination direction of the infrared radiation incident on a sample, and (5) to evaluate if existing preprocessing techniques for retrieving pure absobance spectra from Mie scatter distorted spectra are suitable for correcting infrared spectra of deformed spherical systems.

The dome- and elongated dome-shaped structures were obtained using a 3D-printing technique, namely by two-photon induced direct laser writing (2PDLW).

## Methods

### Infrared transmission measurements

In FTIR spectroscopy, the absorbance spectrum *Z* is determined by measuring $I_0$, the intensity incident on the sample, and *I*, the intensity transmitted by the sample:

1 $$\begin{aligned} Z = -\log _{10} \left( \frac{I}{I_0} \right) , \end{aligned}$$

Ideally, the incoming radiation is only attenuated by molecular absorption, resulting in a pure absorbance spectrum which can be directly interpreted as a molecular fingerprint. However, scattering also contributes to loss of radiation at the detector. The scattering contribution may significantly modify the absorbance spectrum and thus require correction methods to obtain a pure absorbance spectrum^19^.

To calculate the total amount of radiation removed from the forward direction, either by absorption or by scattering, we look at the dimensionless extinction efficiency, $Q_{\textrm{ext}}$, defined as the ratio of the extinction cross section and the geometric cross section of the sample^17^. The following equation shows the relation between the apparent absorbance and the extinction efficiency^19,20^:

2 $$\begin{aligned} Z = -{\log _{10} \left( 1-\frac{g}{G}Q_{ext}\right) }\approx \frac{1}{\ln (10)} \left( \frac{g}{G}\right) Q_{\textrm{ext}}, \end{aligned}$$

where *G* and *g* are the geometric cross sections of the detector and the sample, respectively. In (2) it is assumed that $G\gg g$. Since, according to (2), $Q_{\textrm{ext}}$ features prominently in the absorbance *Z*, $Q_{\textrm{ext}}$ is a quantity of interest. While, except for homogeneous films, there are no exact closed-form analytical expressions available for computing $Q_{\textrm{ext}}$ as a function of wavenumber, closed-form analytical *approximations* of $Q_{\textrm{ext}}$ can be obtained. Due to this equivalence, the measured apparent absorbance spectrum (Eq. 1) is also called the extinction spectrum.

### Analytical formulas for the extinction efficiency

Following Ref.^17^, approximation formulas for $Q_{\textrm{ext}}$ relevant for the simulations and experiments discussed in this paper are found by assuming that the incoming radiation, represented as rays, traverses the scatterer straight-through, without deflection, and thus experiences only a phase shift inside the scatterer. At the detector the incident radiation combines with the phase-shifted radiation resulting in wave interference, which produces $Q_{\textrm{ext}}$. Based on this interference effect, analytical extinction formulas for hemispheres, semi-capsules and semi-cylinders are derived in Appendix A.

We point out that including tunneling and diffraction effects, vast improvements of the accuracy of the formulas presented in the Appendix can be achieved (see, e.g., Ref.^21^ for the case of spheres). These more complex formulas, however, are not needed for the purposes of this paper.

### 3D printing of samples

In two-photon induced direct laser writing (2PDLW), local polymerization is obtained in the very small volume of a tightly focused laser beam upon two-photon absorption^22^. The displacement of the focal spot along a trajectory inducing polymerization of the photoresist results in a solid 3D microstructure. The two-photon absorption process and the subsequent confined polymerization may give rise to 100 nm or even higher fabrication resolution^23^. Such a resolution makes 2PDLW ideally suited for the fabrication of the domes and elongated domes with well-defined size, shape and relative positioning for infrared spectroscopy in controlled conditions.

**Figure 1:**
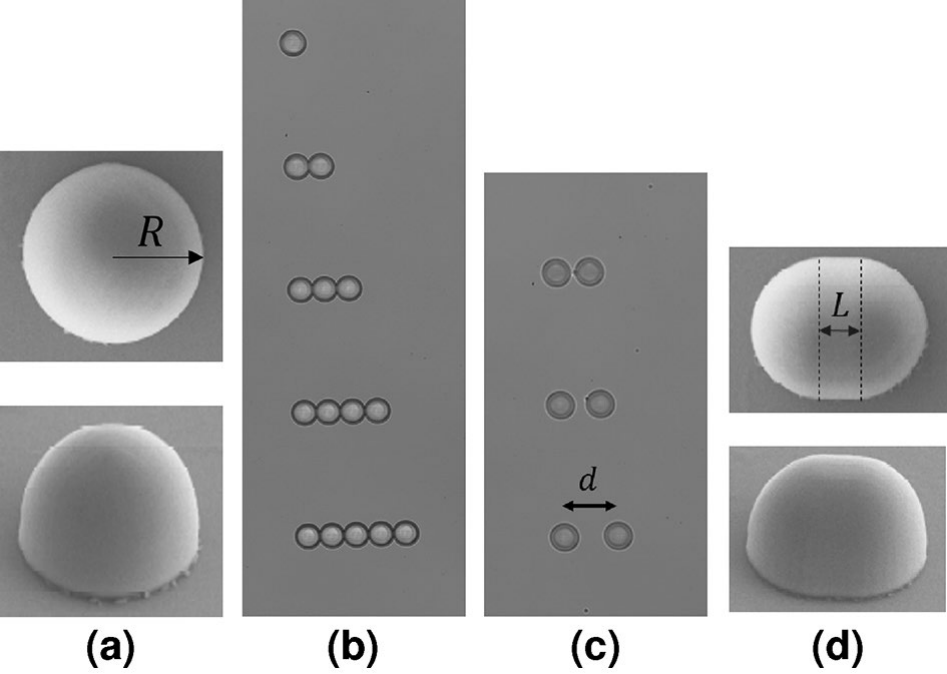
SEM- and microscope images of one of each of the configurations investigated by FTIR spectroscopy. (**a**) A single dome of radius $R=10\,\upmu \hbox {m}$, (**b**) touching domes in a row, (**c**) two domes with increasing distance *d*, and (**d**) semi-capsules (elongated domes) with a radius of the end-caps equal to $R= 10\,\upmu \hbox {m}$ and elongation *L*. The material of the samples is OrmoComp^24^.

The 3D-printed dome-shaped structures were obtained using a commercial 2PDLW setup (Microlight3D). It consists of an inverted microscope (Zeiss Axiovert 200) equipped with a frequency-doubled Q-switched Nd:YAG laser (532 nm, 0.56 ns pulse duration, 11.7 kHz). A 40x objective (NA 0.95, dry, Zeiss Plan-APOCHROMAT) was used to focus the laser beam in the sample. The 3D-objects are obtained by displacing the sample relative to the laser focal spot using a $100\times 100\times 100 \,\upmu \hbox {m}$ travel range piezo manipulator. The laser power can be controlled by an acousto-optic modulator. The piezo manipulator is combined with a long-range translation stage for automatic replication of the structures on the surface of a substrate. An autofocus system is used to position each object at the same position relative to the substrate surface with a reproducibility better than 50 nm. The scan speed, the laser power and the 3D trajectory are computer controlled.

The 3D structures were created using the Ormocomp photoresist (Microresist Technology)^24^. A ca.5 $\upmu \hbox {L}$ drop of photoresist was deposited on the surface of a 170 $\upmu \hbox {m}$ microscope coverslip. After microfabrication the unreacted photoresist was removed by two successive washing steps using Ormodev (Microresist Technology) as a solvent. After the washings, the samples were dried in air. No pre- or postbaking procedure was used in the 3D-printing.

The microfabricated structures were characterized by Scanning Electron Microscopy (SEM) using a Zeiss Supra 55VP apparatus. Prior to SEM experiments, a 5 nm gold coating was deposited on the surface of the samples with a Leica EM ACE600 sputter coater to remove charge accumulation and improve contrast in the SEM measurements.

**Figure 2:**
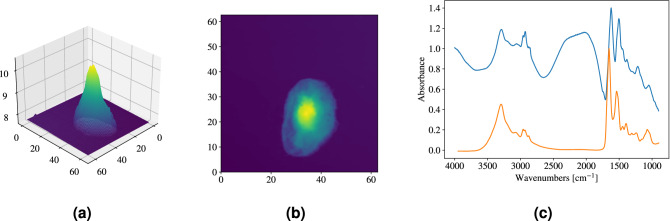
Properties of a lung-cancer cell. (**a**) AFM height map showing its 3D topography, which resembles a dome. (**b**) 2D contour heat map showing the same information as (**a**), but in 2D cross sectional information is brought out more clearly. (**c**) Infrared spectrum of the lung-cancer cell^15,27^ (blue line), containing Mie-scattering contributions, compared with a pure Matrigel absorbance spectrum (orange line), approximately representative of a human cell, devoid of any Mie-scattering contributions.

**Figure 3:**
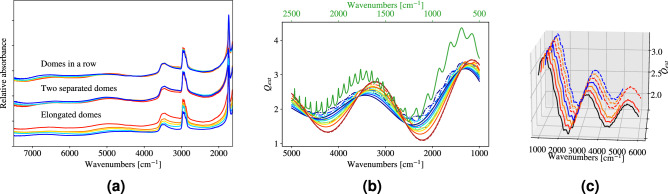
(**a**) Infrared absorbance spectra from dome and elongated dome samples. The mean of 4-5 replicas is shown for each configuration. In all situations the material of the domes is OrmoComp^24^ and the radius of the domes is $10\,\mu$m. The upper set shows absorbance spectra for domes in a row, from a single dome (red line) to five domes in a row (blue line). The middle set shows domes with increasing distance $d = [0, 5, 10, 20]\, \upmu \hbox {m}$ (from red to blue line respectively). The lower set shows absorbance spectra for elongated domes, where the elongation *L* = [0, 2.5, 5, 10, 15] $\upmu \hbox {m}$ (**b**) Extinction efficiency for an elongated dome with refractive index 1.5 for increasing elongation *L*. The radius of the end-caps is *R* = $10\,\upmu \hbox {m}$. The dash-dotted lines show $Q_{\textrm{ext}}$ found by electromagnetic SIE-simulations for cases where *L* is increased from 0 to 3*R*. The solid lines show the approximations according to Eq. (A.16) where *L* is increased from 0 to 50*R*. The red dashed line is the approximation of $Q_{\textrm{ext}}$ for an infinitely long half cylinder (Eq. A.15). The green line shows $Q_{\textrm{ext}}$ for a sphere of radius $10\,\upmu \hbox {m}$ and refractive index of 1.5, computed via exact Mie theory. The green line is associated with the upper (green) wavenumber axis. (**c**) Simulated $Q_{\textrm{ext}}$ for a system consisting of domes in a row (solid lines) and domes with increasing distance (dashed lines). The black line is $Q_{\textrm{ext}}$ for a single dome. The number of domes ranges from one (red line) to four (blue). The wave number region is reduced for the longest array to reduce simulation time. For the domes with increasing distance, the distance *d* between the two domes is increased from 0 to *R*. For both investigations *R* is set to $10\,\upmu \hbox {m}$ and the refractive index is 1.5. The lines are separated horizontally since they have a high degree of overlap. Only small differences in the ripple structure are observed between the lines.

**Figure 4:**
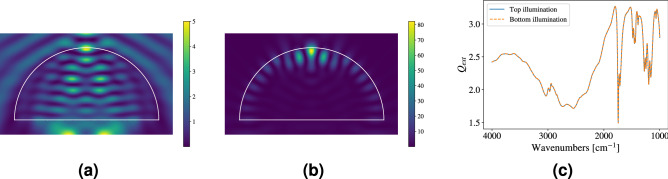
Electric-field distribution $|E|^2$ inside and outside of a dome with $n=1.5$ and $R=10\,\mu$m at $2374\,\textrm{cm}^{-1}$. (**a**) Top illumination. The internal electric-field intensity has the form of horizontal stripes. (**b**) Bottom illumination. The internal electric-field intensity manifests as a whispering-gallery mode. Thus, although $Q_{\textrm{ext}}$ is the same for both illumination directions, the corresponding electric-field distributions inside of the dome are vastly different for top vs. bottom illumination. (**c**) Extinction efficiency for an absorbing dome, with top and bottom illumination. The refractive index of the dome is set to PMMA, as presented in^37^. The extinction efficiency is identical for top and bottom illumination.

The 3D structures obtained using a CAD software were sliced by Simpoly 4.5.1. The internal part of the objects was less densely sliced than the surface to decrease the processing time. The slicing direction was chosen to be perpendicular to the beam propagation axis and the voxel positions were determined following the geometric slope of the structure to improve the surface quality similarly as proposed by Liao et al.^25^. The slicing parameters, exposure time and laser power were varied making numerous replications in feedback with SEM experiments to find the optimal fabrication conditions. Figure 1 shows SEM pictures of the samples which were investigated: (1) domes (Fig. 1a), (2) linear arrays of touching domes (Fig. 1b), (3) domes with increasing distance (Fig. 1c), and (4) semi-capsules (elongated domes) (Fig. 1d).

Dome-shaped (hemi-spherical) and elongated, dome-shaped (semi-capsule) structures were designed using a CAD software. The electron microscopic and IR optical microscopic images of the structures are shown in Fig. 1. The radius of the domes was $10\,\upmu \hbox {m}$. The elongated domes (semi-capsules) were designed by combining two quarter spheres placed at a distance *L* and hemi-cylinder ($R=10\,\upmu \hbox {m}$ and increasing lengths, *L*) placed between them (see Fig. 1d). The radius was kept constant, but *L* was varied from 0 to 1.5*R* in the study. Besides fabricating isolated individual domes, they were replicated at various relative positions to each other to study their couplings as shown in Fig. 1b and c. Two types of configurations were used. First, linear arrays of domes were fabricated positioning them one beside the other. The number of domes in an array was varied from two to five. Then, pairs of domes were fabricated at relative spacing *d*, varied between 0 and 2*R*. All configurations were fabricated in replicates of 5.

### Infrared measurements

#### AFM measurements of lung cancer cells

Atomic force microscopy images were collected using a JPK NanoWizard II AFM (JPK, Cambridge, UK) mounted on a Zeiss AxioObserver, in contact mode. Silicon nitride cantilevers (Veeco, Cambridge, UK) were used.

#### FTIR measurements of lung cancer cells

FTIR spectra from a lung-cancer cell line (CALU-1) were recorded using a synchrotron-coupled Nicolet Continuum IR microscope (ThermoFisher Scientific, Courtaboeuf, France), with a 32$\times$ Schwarzschild objective and an MCT single-element detector. The measurements were performed at the SMIS beamline at the SOLEIL synchrotron, France. Details about the experiment can be found in ref.^26^. In this experiment, cells were obtained from the non-small cell lung cancer (NSCLC) cell line SK-MES purchased from the European Collection of Cell Cultures (Salisbury, U.K.)^20,27^.

#### FTIR Measurements

FTIR-spectra of 3D-printed domes and semi-capsules were collected at the SMIS beamline at the SOLEIL synchrotron. All samples were measured with the same FTIR microscope as the lung cancer cells, with the same magnification and detector. The confocal aperture was set to $10\times 10\,\upmu \hbox {m}$ for the domes and for the semi-capsules. The width of the aperture was increased in concert with *L* to cover the entire spatial extent of the semi-capsule. The spectral resolution was set to 4 $\hbox {cm}^{-1}$. A total of 64 scans were averaged for each sample, and 512 for the background. Spectra were collected from four to five replicas for each configuration. The average of the spectra for each of the configurations was then calculated.

### Numerical investigations

The simulations were performed by the surface integral equation (SIE) method^28^. This is a powerful numerical method which is frequently used to solve three-dimensional electromagnetic problems. The SIE method solves the equivalent surface-current densities on the surface of the sample, and thereby reduces the number of unknowns compared with volume-based methods (such as FEM, FDTD, or VIEM)^29^. There are various SIE formulations for homogeneous dielectric objects. In our code we use the combined tangential formulation (CTF). The Multilevel Fast Multipole Algorithm (MLFMA)^30^ is employed to further improve the efficiency of the SIE solution. The extinction efficiency is calculated from the SIE solution vector and the excitation vector^31,32^. Our code has been validated by the analytical Mie solution^33^ and has been used for the simulation of the homogeneous dielectric object in our previous work^33^.

## Results and discussion

### AFM and infrared measurements of lung-cancer cells

In general, as a result of investigating the shapes of biological samples, it is evident that a biological cell may not resemble a perfect sphere when deposited onto a substrate. An example of an AFM height map, showing the topography of a lung-cancer cell, is shown in Fig. 2a. The figure shows that the shape of the cell is closer to a dome than to a sphere. The lung-cancer cell sticks to the surface of the slide, and the shape is therefore deformed. This strengthens our assumption that in many cases of structurally soft samples, dome-shaped systems are better model systems for describing biological cells. A 2D contour heat map (Fig. 2b) shows the same information as in Fig. 2a, but the cell’s structure in 2D is quantitatively better represented. An infrared spectrum collected from the same cell is shown in Fig. 2c, where the scattering signatures are clearly present^15,27^. In contrast, in the same figure, a representative spectrum for human cells (represented by a Matrigel spectrum^15^), devoid of scattering features, is shown in orange. The pure absorbance spectrum (orange line), is approximately what we would expected to measure in the case where the cancer cell is non-scattering.

### Infrared measurements of 3D-printed systems

Three different systems were investigated, i.e., domes in a row (Fig. 1b), two domes with increasing distance (Fig. 1c), and semi-capsules for varying elongation *L* (Fig. 1d). The spectra of these systems are reported in Fig. 3a. All spectra in Fig. 3 are the mean of spectra from identical systems.

We start with domes in a row. As shown in Fig. 1b, the number of domes ranges from one to five, where the single dome serves as the reference system (no neighbors) and the domes in rows two to five are all touching for maximal coupling between domes. In each row, we recorded the absorbance spectrum from one of the domes close to the center of the row. The resulting absorbance spectra are shown in the upper set in Fig. 3a. We observe that despite the proximity of the domes, and independent of the number of touching domes in a row, the effect of neighboring domes on the spectra is negligible. The slight variations in the scattering signatures, most pronounced in the region from 7500–5000 cm$^{-1}$, are not systematic.

Figure 3a shows absorbance spectra of two domes of increasing distance (middle set) as shown in Fig. 1c. The measurements are done on one of the domes in the pair. Figure 3a shows that the variations in absorbance are negligible for this system as well. The red line is the absorbance for touching domes. The other lines show the absorbance for increasing distance *d* between the domes, up to $d=2R$ (blue line).

Investigating semi-capsules, where *L* in Fig. 1d is gradually increased from 0 to 1.5*R*, we see that the wiggle structure is shifted towards lower wavenumbers (lower set in Fig. 3a). The vertical shift in absorbance seen in these spectra, as described in Methods, is caused by the changing size (increased in concert with *L*) of the numerical aperture.

### Simulations of the extinction efficiency

The extinction efficiency $Q_{ext}$ is related to the measured absorbance by Eq. (2). As mentioned above, for special systems with a high degree of symmetry, the exact extinction efficiency can be found analytically from electromagnetic theory, e.g., Mie Theory^34^ in the case of spheres. In this case we neglect the presence of the microscope slide. This effect will be investigated in a follow-up paper. For spheres, we can also find the approximate analytical solution (see Appendix A). However, for non-integrable systems, where no exact analytical results can be found, only the approximate approach can be taken for the analytical solution. We can also use numerical approaches such as SIE to solve the scattering problem of the non-integrable systems.

Since a dome has axial symmetry but does not have spherical symmetry, top illumination (the incident beam propagates in the negative *z* direction, i.e., the incident radiation enters the tip of the dome toward the bottom surface of the dome) can be distinguished from bottom illumination (the incident beam propagates in the positive *z* direction, i.e., the incident radiation enters through the bottom surface of the dome toward the tip of the dome). We checked numerically that top and bottom illumination yield the same $Q_{\textrm{ext}}$, which is guaranteed by the optical reciprocity theorem^35^ (see Fig. 4c).

Figure 3b shows the analytical Mie solution in green, together with the approximate $Q_{\textrm{ext}}$ for elongated domes according to Eq. (A.16) in solid lines, and numerical simulations for $Q_{\textrm{ext}}$ for elongated domes in dash-dot lines. Note that the analytical Mie solution correspond to the upper green *x*-axis. For the domes, the elongations vary from no elongation ($L=0$, blue lines) to $L=$ [ 2.5, 5, 10, 15, 30 ] $\upmu \hbox {m}$ , where the longest elongation corresponds to the red lines. The radius of the domes is set to *R*=$10\,\upmu \hbox {m}$, and the refractive index to 1.5, which is approximately the refractive index of OrmoComp^24^ blue if absorption is disregarded. By simulating a non-absorbing particle, we isolate the effect of scattering on $Q_{\textrm{ext}}$. The first thing we notice from Fig. 3b is that the wiggles in $Q_{\textrm{ext}}$ coincide for the numerical calculations (dash-dotted lines) and the approximation formula (solid lines).

In addition to the approximation of $Q_{\textrm{ext}}$ for an elongated dome with semi-cylinder elongation $L=500\,\upmu \hbox {m}$ (dark red solid line), Fig. 3b shows $Q_{\textrm{ext}}$ for a semi-cylinder (Eq. A.15) as the dashed dark red line. It is evident that for increasing *L*, the resulting $Q_{ext}$ approaches the one of a semi-cylinder. The numerical calculations of $Q_{\textrm{ext}}$ exhibit the same trend as the approximated $Q_{\textrm{ext}}$, however an elongation of $L=500\,\upmu \hbox {m}$ is too large for numerical calculations to be made.

The numerical simulations are done for an incident field which is polarized such that the electric field is perpendicular to the elongated axis of the semi-cylinder. As is evident from the figure, the elongation causes the ripples to weaken. This is because the whispering gallery modes are strongest in the direction perpendicular to the electric field, which is the axis of elongation. However, when $L>0$, these whispering gallery modes die out since the deformed shapes do not support standing waves along the boundary. This is corroborated by the results reported in reference^20^. For polarization of the electric field parallel to the elongated axis, see Appendix B.

From Fig 3b it is further evident that the wiggles in the approximate Qext and the numerical Qext also coincide with the full Mie solutions when one corrects for the factor-2 reduced path length of infrared rays traversing a dome compared to the path length required to transverse a sphere. The purpose of showing this is to demonstrate the relation between Qext for a full sphere and a dome. We note that in the case of dispersion, a scaling due to the wavelength dependence of the refractive index would be required as well. The ME-EMSC model, however does not require a prior knowledge of the scaling and adjusts the scaling of the wiggles and dispersion automatically. Therefore, the results support the applicability of the van-de-Hulst approximation for the correction of spectra obtained from dome-like scatterers.

Although much weaker, Fig. 3b shows that the ripples in the numerical dome $Q_{\textrm{ext}}$ (dash-dotted blue line) line up with the ripples in the sphere $Q_{\textrm{ext}}$ (green line). This is understood on the basis of the fact that the discontinuity in the index of refraction at the bottom surface of the dome, i.e., transitioning abruptly from $n=1.5$ on the inside of the dome to $n=1$ on the outside of the dome, acts like an imperfect mirror that is capable of complementing dome resonances into sphere resonances via their mirror image. Thus, any resonance in the sphere with up/down symmetry is also a dome resonance. The reduced contrast of dome ripples vs. sphere ripples is also understood, since the bottom-surface mirror of the dome is imperfect (i.e., apart from reflection, it also allows significant transmission), the dome resonances can easily leak out of the dome volume through the mirror surface, and thus result in significantly reduced prominence of dome ripples. Because of the leak of radiation through the bottom surface of the dome, a dome can also be viewed as a leaky dielectric resonator. Any leaks in a resonator reduce the quality factor of the resonator, resulting in broadening of resonances. This mechanism explains why the observed dome ripples are not only smaller in height, but also much broader than the corresponding sphere ripples.

We also performed numerical simulations of $Q_{ext}$ for touching domes in a row (Fig. 1b). The results from these simulations are shown in Fig. 3c as solid lines. It is evident that the change in $Q_{ext}$ for the different systems is small. The same is true for two domes with increasing distance (Fig. 1c), whose $Q_{ext}$ is plotted in Fig. 3c as dashed lines. The distance *d* between the domes is varied from approximately no distance ($d=0.04\,\upmu \hbox {m}$) to $d=R=$ $10\,\upmu \hbox {m}$. $Q_{ext}$ is practically not affected. These results corroborate what is reported in references^33,36^, which shows that the same conclusions can be drawn for small aggregates of spheres, both touching and separated. Reference^33^ also shows that for an aggregate of spheres with different radii, the extinction efficiency takes on the mean from all spheres individually. One can assume that the same conclusion holds for hemispheres.

In Appendix  C, the results of similar numerical investigations are reported for two-dimensional systems consisting of half-disk-shaped scatterers and half-stadium-shaped scatterers. These systems are equivalent to three-dimensional systems which are invariant in the third dimension (e.g., infinitely long semi-cylinders). The simulations show the same trend as the three-dimensional results, i.e., the effect of neighboring scatterers is negligible but the transition from a half-disk to a half-stadium produces a shift of the wiggles in the extinction efficiency.

### Top vs. bottom illumination

In the previous section we pointed out that the optical reciprocity theorem^35^ requires only that $Q_{\textrm{ext}}$ is invariant with respect to bottom and top illumination (as showed in Fig. 4c), but is silent on the shape of the internal electric-field distributions of the scatterer in these two cases. Indeed, bottom illumination yields a different electric-field intensity distribution than top illumination. Simulations were performed with both top and bottom illumination, for a dome with $R=10\,\mu$m. A complex refractive index was employed to demonstrate that the invariance in $Q_{\textrm{ext}}$ holds for absorbing materials as well. The refractive index was set to PMMA^37^, since the refractive index of OrmoComp remains unknown. As a result of our detailed numerical simulations, Fig. 4a shows the electric-field intensity distribution for a dome using top illumination, while Fig. 4b shows the electric-field intensity distribution for the same dome but with bottom illumination. We obtain completely different electric-field distributions in these two cases. In fact, while top illumination yields an intensity distribution in the form of horizontal stripes (Fig. 4a), bottom illumination yields an intensity distribution in the form of a whispering-gallery mode^18^ (Fig. 4b). Whispering-gallery modes were identified before as the origin of the ripples in the $Q_{\textrm{ext}}$ of spheres (see the sharp features in $Q_{\textrm{ext}}$ shown in Fig. 3b). As explained in the previous section, and since whispering-gallery modes are up/down symmetric, we should expect them to occur in domes as well, since the imperfect bottom-surface mirror can complete the whispering-gallery arc shown in Fig. 4b into a complete whispering-gallery mode of a sphere and explain the ripples and their positions at the same locations as sphere ripples in the case of domes. The surprise is that in the case of domes the morphology of the corresponding resonance wavefunctions depend on the illumination direction. The different electric-field distribution in dome-shaped scatterers (and other irregularly shaped scatterers) may have important consequences for infrared spectroscopy, since, as shown in Fig. 4 different internal regions of the scatterer are irradiated with different intensities depending on top vs. bottom illumination. For instance, considering a cell in the form of a nested dome, bottom illumination would preferentially probe the cell wall, while top illumination would preferentially co-probe the internal volume of the cell. Thus, a modest degree of spatially resolved cell chemistry could be obtained. While these differences, because of the optical reciprocity theorem, cannot be seen in $Q_{\textrm{ext}}$, they might be seen if in addition to the transmitted infrared radiation the scattered radiation is also measured. This is partially done with the numerical aperture in infrared microscopes. We have not yet performed experiments addressing top vs. bottom illumination, but our detailed SIE-simulations (backed up with some independent, preliminary VIEM-simulations) are quite clear on the different internal-field distributions (see Fig. 4) and the corresponding different scattering signatures.

The conclusions we drew in the case of domes can be backed up explicitly in the case of a film stack with two absorbing layers. In this case closed-form analytical expressions can be obtained for all relevant quantities, such as extinction, scattering, and absorption efficiencies, i.e., $Q_{\textrm{ext}}$, $Q_{\textrm{scat}}$, and $Q_{\textrm{abs}}$, respectively. The formulas confirm optical reciprocity^35^ by analytically obtaining the same $Q_{\textrm{ext}}$, even in the absorbing case. They also show that the internal electric-field distribution is different in the two films for top vs. bottom illumination, which, in the case of the absorbing film stack can be computed explicitly and analytically.

We also performed additional numerical simulations with semi-cylinders and semi-capsules confirming that, while $Q_{\textrm{ext}}$ is invariant, top vs. bottom illumination for these systems as well produces different internal electric-field intensity distributions for top vs. bottom illumination.

In the context of top vs. bottom illumination, one may wonder about oblique incidence. We have not investigated this situation further, but most likely the same effects will happen. However, in the case of oblique incidence the polarization of the incident beam matters and the reciprocal conditions have to be carefully defined. While even in the case of oblique incidence $Q_{\textrm{ext}}$ is still expected to be invariant, we do expect that the internal electric-field distribution is again different. These are difficult but important questions that will be the subject of further research.

### Using van-de-Hulst sphere solutions of $Q_{ext}$ for modelling semi-capsule $Q_{ext}$

In order to estimate and remove scattering signatures from spherical biological samples, the ME-EMSC algorithm^15^ is considered state-of-the-art. The algorithm is based on a meta model which employs the van-de-Hulst approximation to $Q_{ext}$ for a sphere. Several plausible solutions for $Q_{ext}$ are calculated, which are then compressed to a number of basis vectors. The basis vectors are used to reconstruct the measured absorbance spectrum. As pointed out in this paper, biological samples are rarely perfectly spherical, and can approximate, for example, domes or semi-capsules. It is therefore important to know whether the $Q_{ext}$ of a semi-capsule exists within the subspace of basis vectors spanned by the sphere solutions which are used in the meta model. While it is clear that the $Q_{ext}$ of a hemisphere exists within this subspace, since the solutions for the van-de-Hulst approximations are identical for a sphere and a hemisphere (adjusting for the difference in effective optical path length), this is not the case for a semi-capsule where $L>0$.

**Figure 5:**
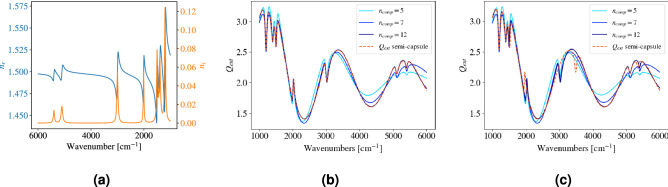
(**a**) The refractive index used in the calculations of $Q_{\textrm{ext}}$, where the real part $n_r$ is shown in blue and the imaginary part *n* is shown in orange. (**b**) $Q_{\textrm{ext}}^{\mathrm{semi-capsule}}$ (red dashed line) is reconstructed from a number $n_{comp}$ basis vectors. The blue lines show different reconstructed $Q_{\textrm{ext}}^{\mathrm{semi-capsule,r}}$, with a different number of basis vectors used. (**c**) When the chemistry (determined through the refractive index) used for calculating the 100 $Q_{\textrm{ext}}^{\textrm{sphere}}$ curves is changed, the reconstruction $Q_{\textrm{ext}}^{\mathrm{semi-capsule,r}}$ fails at the absorption bands.

To answer this question, we consider an absorbing semi-capsule whose refractive index is shown in Fig. 5a. The real part of the refractive index is shown in blue, and the imaginary part in orange. The refractive index is established by simulating Lorentz lines for the imaginary part, and using the Kramers-Kronig relations to calculate the fluctuating real part of the refractive index. A constant offset of 1.5 for the real part is used. A simulated refractive index is used such that the absorption properties of the material can be varied. The extinction efficiency for a semi-capsule $Q_{\textrm{ext}}^ \mathrm{semi-capsule}$ with $R =10\,\upmu \hbox {m}$ and $L = 10\,\upmu \hbox {m}$ is shown as the dashed red line in Fig. 5b. Equation (A.12) is used to calculate the semi-capsule $Q_{\textrm{ext}}^ \mathrm{semi-capsule}$.

100 extinction efficiencies $Q_{\textrm{ext}}^{\textrm{sphere}}$ for absorbing spheres were then calculated, according to Eq. (A.2). The complex refractive index was the same as for the semi-capsule, only that the constant offset for the real part was varied between 1.3 and 1.7. The radii of the spheres were varied from $R = 5\,\upmu \hbox {m}$ to $R = 10\,\upmu \hbox {m}$. Further, the curves are compressed by PCA to a number $n_{\textrm{comp}}$ of principal components, $p_i$, as in the ME-EMSC meta model^15^.

In order to find if $Q_{\textrm{ext}}^ \mathrm{semi-capsule}$ can be expressed in terms of the $p_i$, $Q_{\textrm{ext}}^ \mathrm{semi-capsule}$ was projected onto the space spanned by $p_i$. The reconstructed $Q_{\textrm{ext}}^ {\mathrm{semi-capsule}, r}$ are shown together with the original $Q_{\textrm{ext}}^ \mathrm{semi-capsule}$ in Fig. 5b. The reconstructed $Q_{\textrm{ext}}^{\mathrm{semi-capsule}, r}$ are established with a different number of principal components $n_{\textrm{comp}}$, and are shown in different shades of blue. The original $Q_{\textrm{ext}}^{\mathrm{semi-capsule}}$ is shown in dashed red. It is evident that for a small number of principal components ($n_{\textrm{comp}} = 5$), the reconstruction has a relatively large error. When increasing the number of principal components, the reconstruction improves. For $n_{\textrm{comp}} = 12$, the reconstruction is almost perfect. For longer *L*, an increasing number of principal components are generally needed to obtain a satisfactory reconstruction (results not shown). However, for $n_{\textrm{comp}} > 9$, the reconstruction is also satisfactory for long elongations.

In the ME-EMSC, the number of principal components used in the subspace model is a variable that needs to be set by the user. The default value is based on the explained variance, and is usually 7. Our results show that for non-spherical samples, it is expected that the model still describes $Q_{\textrm{ext}}$ for deformed spheres. However, an increase in the number of components in the model might be needed. In this analysis we show that the sphere-subspace model employed in the ME-EMSC also contains the solution for an elongated dome. We therefore conclude that the ME-EMSC is also valid for elongated, dome-shaped systems, which is a more representative model of a biological cell.

To demonstrate that the $p_i$ cannot reconstruct $Q_{\textrm{ext}}^{\mathrm{semi-capsule}}$ calculated from an arbitrary complex refractive index, a slightly different chemistry was simulated and used as input for $Q_{\textrm{ext}}^{\mathrm{semi-capsule}}$. The difference from the original complex refractive index is that some peak positions were moved according to: 1500 $\hbox {cm}^{-1}$ $\rightarrow$ 1520 $\hbox {cm}^{-1}$, 2010 $\hbox {cm}^{-1}$ $\rightarrow$ 1950 $\hbox {cm}^{-1}$, 3000 $\hbox {cm}^{-1}$ $\rightarrow$ 3500 $\hbox {cm}^{-1}$, 5400 $\hbox {cm}^{-1}$ $\rightarrow$ 5350 $\hbox {cm}^{-1}$. When reconstructing the new $Q_{\textrm{ext}}^{\mathrm{semi-capsule}}$ with the $p_i$ from before, the shifted peaks cannot be accurately retrieved. Figure 5c shows the reconstructed $Q_{\textrm{ext}}^{\mathrm{semi-capsule}, r}$ in shades of blue together with the new $Q_{\textrm{ext}}^{\mathrm{semi-capsule}}$ in dashed red. It is evident that the reconstructed spectra fail to describe the shifted peaks.

## Conclusion

By evaluating the AFM-image of a lung-cancer cell (see Fig. 2a), we observe that its shape is more consistent with a dome than a sphere. Similar to lung-cancer cells, this morphology is expected in general for many structurally unstable biological cells when deposited onto microscope slides. As seen in Fig. 2c, we still observe strong scattering features from such dome-shaped systems. This is corroborated by our numerical investigations, which show strong Mie scattering signatures from dome-shaped systems. In fact, disregarding the ripples, we expect that domes exhibit exactly the same wiggle structure as perfect spheres when correcting for the difference in effective optical path length (i.e., factor-2 scaling of $\rho$). For increasing *L* we observe that elongated domes show a wiggle structure very similar to spheres, with a gradual shift towards lower wavenumbers.

Further, we observe no significant difference in the scattering signatures of isolated domes versus dome arrays. The measurements (Fig. 3a) of the 3D printed samples (Fig. 1) indicate that the effect of coupling between scatteres is negligible. The wiggle structure is practically unchanged, while minor differences can be observed in the ripple structure. We have demonstrated that this conclusion is valid for both our numerical simulations and our measurements. These results are consistent with the results reported in Ref.^33^ on sphere arrays.

This paper contributes to a better understanding of scattering problems with important implications for the models used to correct scattering in these systems. In infrared spectroscopy, the ME-EMSC algorithm is the state-of-the-art preprocessing method used for removing Mie scattering signatures from spectra of biological cells and tissues, thereby retrieving the corresponding pure absorbance spectra^15^. The algorithm employs a meta model based on the van-de-Hulst approximation for scattering off a sphere to model the scattering features in the measured absorbance spectra. Based on the results reported in this paper, we conclude that a model which assumes the scatterer to be a perfect sphere is applicable for dome- and semi-capsule-shaped systems as well. This has been demonstrated by theoretical considerations, proving that the theory behind the ME-EMSC model is applicable for dome- and semi-capsule systems. Further, since coupling effects between systems of multiple spheres and domes are negligible, the ME-EMSC model is also applicable to biological cells in arrays.

When reporting absorbance spectra, the illumination direction (i.e., top vs. bottom illumination) of biological samples in infrared microspectroscopy is not usually specified in the literature. And, indeed, the optical reciprocity theorem^35^ guarantees that, as far as $Q_{\textrm{ext}}$ is concerned, reversing the illumination direction yields the same results. However, the determination of $Q_{\textrm{ext}}$ requires that only forward-scattered light is to be recorded in the infrared detector. For any realistic detector, however, this cannot be achieved, since, because of the numerical aperture (NA) of the detector, scattered radiation is always recorded together with the radiation in forward direction. This is a major problem, since the *scattered* radiation depends on the illumination direction. Thus, for any realistic detector, the absorbance measured for two conjugate directions (e.g., top and bottom illumination) of the incident radiation will be different. Spectroscopists need to be aware of this problem, and it has to be properly taken into account in scatter-correction algorithms. This will be an important direction for future work.

## Supplementary Information

Supplementary Information.

## Data Availability

The datasets generated and/or analysed during the current study are available upon request from the corresponding author in the Zenodo repository within the BioSpec Norway Community;https://doi.org/10.5281/zenodo.7228232.
