# Supplementary material for: Domes and semi-capsules as model systems for infrared microspectroscopy of biological cells
Source: Sci Rep. 2023 Feb 23;13:3165. doi: 10.1038/s41598-023-30130-z (PMC9950083; doi:10.1038/s41598-023-30130-z)
Supplement: Supplementary file 1 — Supplementary Information. [file 41598_2023_30130_MOESM1_ESM.pdf]

Appendix

Domes and Semi-Capsules as Model Systems for Infrared Microspectroscopy of Biological Cells

A Analytical Q_{ext} Formulas

In this appendix, for convenience, we state the known analytical extinction formula for spheres[17] and, building on the results and methods in Ref.[17], derive analytical extinction formulas for domes, semi-cylinders, and semi-capsules. Define the complex index of refraction

$$n = n_r + in_i, \tag{A.1}$$

where $n_r > 0$ and $n_i \geq 0$ are its real and imaginary parts, respectively. A semi-capsule consists of two quarter-spheres as end-caps and a mid-section in the shape of a semi-cylinder. The two quarter-sphere end-caps add up to a hemisphere, i.e., a dome. According to Ref.[17], Q_{ext} for a sphere is

$$\begin{aligned} Q_{\text{ext}}^{\text{sphere}} &= \Re\left\{2 - \left(\frac{4i}{\hat{\rho}}\right)\exp(-i\hat{\rho}) + \left(\frac{4}{\hat{\rho}^2}\right)[1-\exp(-i\hat{\rho})]\right\} \\ &= \Re\left\{2 - \left(\frac{4}{\hat{\rho}}\right)\sin(\hat{\rho}) + \left(\frac{4}{\hat{\rho}^2}\right)[1-\cos(\hat{\rho})]\right\} \\ &= 2 - 4e^{-\rho\tan(\beta)}\left(\frac{\cos(\beta)}{\rho}\right)\sin(\rho-\beta) \\ &\quad - 4e^{-\rho\tan(\beta)}\left(\frac{\cos(\beta)}{\rho}\right)^2\cos(\rho-2\beta) + 4\left(\frac{\cos(\beta)}{\rho}\right)^2\cos(2\beta), \end{aligned} \tag{A.2}$$

where

$$\begin{aligned} \hat{\rho} &= \rho[1 - i\tan(\beta)], \\ \rho &= 2x(n_r - 1), \\ x &= 2\pi\tilde{\nu}R, \\ \tan(\beta) &= \frac{n_i}{n_r - 1}, \end{aligned} \tag{A.3}$$

$\tilde{\nu}$ is the wavenumber, x is the size parameter, R is the radius of the sphere, and $\Re$ indicates the real part. Since ρ is the phase shift experienced by a ray of infrared radiation traversing a full sphere, we obtain Q_{ext} for a dome immediately from (A.2) with the substitution $\rho \to \rho/2$, which implies $\hat{\rho} \to \hat{\rho}/2$. Explicitly, the result is

$$\begin{aligned} Q_{\text{ext}}^{\text{dome}} &= \Re\left\{2 - \left(\frac{8i}{\hat{\rho}}\right)\exp(-i\hat{\rho}/2) + \left(\frac{16}{\hat{\rho}^2}\right)[1-\exp(-i\hat{\rho}/2)]\right\} \\ &= \Re\left\{2 - \left(\frac{8}{\hat{\rho}}\right)\sin(\hat{\rho}/2) + \left(\frac{16}{\hat{\rho}^2}\right)[1-\cos(\hat{\rho}/2)]\right\} \\ &= 2 - 8e^{-\rho\tan(\beta)/2}\left(\frac{\cos(\beta)}{\rho}\right)\sin[(\rho/2)-\beta] \\ &\quad - 16e^{-\rho\tan(\beta)/2}\left(\frac{\cos(\beta)}{\rho}\right)^2\cos[(\rho/2)-2\beta] + 16\left(\frac{\cos(\beta)}{\rho}\right)^2\cos(2\beta). \end{aligned} \tag{A.4}$$

For the computation of $Q_{\text{ext}}^{\text{semi-capsule}}$, we also need the extinction cross section $C_{\text{ext}}^{\text{dome}}$, which is obtained from (A.4) by multiplying with the geometric cross section of the dome, i.e.,

$$C_{\text{ext}}^{\text{dome}} = \pi R^2 Q_{\text{ext}}^{\text{dome}}, \tag{A.5}$$

where $Q_{\text{ext}}^{\text{dome}}$ is defined in (A.4). Next, we compute the extinction cross section of a semi-cylinder with radius R and length L. According to Ref.[17] we have

$$C_{\text{ext}}^{\text{semi-cylinder}} = \frac{4\pi}{k^2}\Re\left[S^{\text{semi-cylinder}}(0)\right], \tag{A.6}$$

where $k = 2\pi\tilde{\nu}$ and

$$S^{\text{semi-cylinder}}(0) = \frac{k^2}{2\pi}\int\int\left[1 - e^{-i(\hat{\rho}/2)\sin(\tau)}\right]d\xi d\eta \tag{A.7}$$

is the scattering amplitude of the semi-cylinder in forward direction (i.e., the direction orthogonal to its axis), τ is the angle subtended by an infrared ray piercing the semi-cylinder[17], the integration is over the geometric cross section of the semi-cylinder, and ξ, η are the coordinates of the rectangular base-plate of the semi-cylinder. With (A.6) and (A.7), we obtain

$$C_{\text{ext}}^{\text{semi-cylinder}} = 2\Re\left\{2RL - 2L\int_0^R e^{-i(\hat{\rho}/2)\sin(\tau)}d\xi\right\}, \tag{A.8}$$

where

$$\xi = R\cos(\tau). \tag{A.9}$$

With this substitution, we can now write

$$\begin{aligned} C_{\text{ext}}^{\text{semi-cylinder}} &= 4RL\Re\left\{1 - \int_0^{\pi/2}\sin(\tau)e^{-i(\hat{\rho}/2)\sin(\tau)}\,d\tau\right\} \\ &= 2\pi RL\Re\left\{H_1(\hat{\rho}/2) + iJ_1(\hat{\rho}/2)\right\}, \end{aligned} \tag{A.10}$$

i.e.,

$$Q_{\text{ext}}^{\text{semi-cylinder}} = C_{\text{ext}}^{\text{semi-cylinder}}/(2RL) = \pi\Re\left\{H_1(\hat{\rho}/2) + iJ_1(\hat{\rho}/2)\right\}, \tag{A.11}$$

where $H_1(z)$ is the complex Struve function of order 1, defined in Ref.[38], formula 12.1.5, and $J_1(z)$ is the complex Bessel function of order 1, defined in Ref.[38], formula 9.1.10.

We can now combine these results to obtain

$$Q_{\text{ext}}^{\text{semi-capsule}} = \frac{C_{\text{ext}}^{\text{dome}} + C_{\text{ext}}^{\text{semi-cylinder}}}{\pi R^2 + 2RL}, \tag{A.12}$$

where $C_{\text{ext}}^{\text{dome}}$ is defined in (A.5) and $C_{\text{ext}}^{\text{semi-cylinder}}$ is defined in (A.10).

For real refractive index ($n_i = 0$), the Q_{ext} formulas simplify considerably. In this special case, we obtain:

$$Q_{\text{ext}}^{\text{sphere}} = 2 - \frac{4}{\rho}\sin(\rho) + \frac{4}{\rho^2}[1 - \cos(\rho)]., \tag{A.13}$$

$$Q_{\text{ext}}^{\text{dome}} = 2 - \frac{8}{\rho}\sin(\rho/2) + \frac{16}{\rho^2}[1 - \cos(\rho/2)], \tag{A.14}$$

$$Q_{\text{ext}}^{\text{semi-cylinder}} = \pi H_1(\rho/2), \tag{A.15}$$

$$Q_{ext}^{\text{semi-capsule}}(\rho) = \left(\frac{2\pi R}{\pi R + 2L}\right)\left\{1 - \frac{4}{\rho}\sin\left(\frac{\rho}{2}\right) + \frac{8}{\rho^2}\left[1 - \cos\left(\frac{\rho}{2}\right)\right] + \frac{L}{R}H_1\left(\frac{\rho}{2}\right)\right\}. \tag{A.16}$$

According to Ref.[38], formula 12.1.20, we can expand the Struve function H_1 in (A.15) into a Bessel-function series according to

$$H_1(z) = \frac{2}{\pi} - \frac{2}{\pi}J_0(z) + \frac{4}{\pi}\sum_{m=1}^{\infty}\frac{J_{2m}(z)}{4m^2 - 1}. \tag{A.17}$$

Thus, for a cylinder with real index of refraction, we obtain from (A.15) with (A.17) and $\rho/2 \to \rho$:

$$Q_{\text{ext}}^{\text{cylinder}} = 2 - 2J_0(\rho) + 4\sum_{m=1}^{\infty}\frac{J_{2m}(\rho)}{4m^2 - 1}, \tag{A.18}$$

which agrees with the Q_{ext} formula in Ref.[20]. For a semi-cylinder with real refractive index we then obtain

$$Q_{\text{ext}}^{\text{semi-cylinder}} = 2 - 2J_0(\rho/2) + 4\sum_{m=1}^{\infty}\frac{J_{2m}(\rho/2)}{4m^2 - 1}. \tag{A.19}$$

B Simulations of Q_{ext} for semi-capsules with different polarization

Numerical calculations of the scattering efficiency of the semi-capsule system was performed.From these calculations, both the wiggles and ripples in Q_{ext} are represented. As described above, the ripples die out when L increases, in the case where the electric field is perpendicular to the axis of elongation (see Fig. B.1a). This is because the deformation does not support the standing-wave pattern, which is described in[20]. For polarization of the electric field parallel to the axis of elongation, however, the ripples are not suppressed. This can be seen in Fig. B.1b. The reason for this is that the whispering gallery modes are strongest in the direction which is not affected by elongation, i.e. they are not affected by the deformation. Therefore, the whispering gallery modes are induced all along the length of the semi-capsule, perpendicular to the axis of elongation.

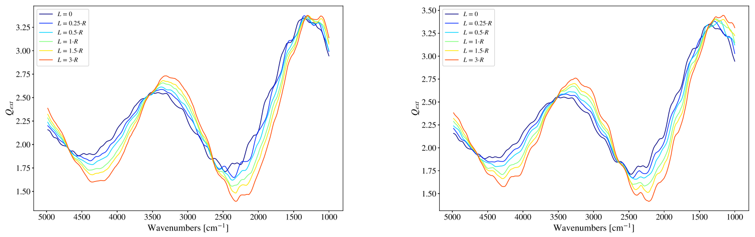

(a) **(b)**

Figure B.1. Q_{ext} for a semi-capsule with increasing elongation L, for (a) electric field polarized perpendicular to the axis of elongation and (b) electric field polarized parallel to the axis of elongation.

C 2D simulations

Equivalent two-dimensional systems were also investigated. The two-dimensional systems correspond to three-dimensional systems that are invariant in the third dimension. In our case, the systems consist of semi-cylinders, where the radii of the semi-cylinders are 10 µm with refractive index 1.5. It is interesting to evaluate systems like this, since, unlike the domes, they have an infinitely large touching surface. The two-dimensional systems were simulated with the help of the Wave Optics Module from COMSOL Multiphysics®[39].

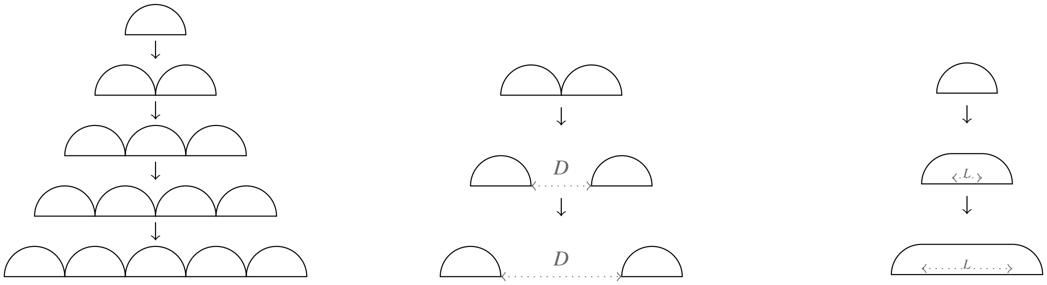

(a) **(b)** **(c)**

Figure C.1. 2D-systems investigated: (a) One up to five semi-cylinders in a row. (b) Two semi-cylinders with increasing distance d. (c) A scatterer which changes from a semi-cylinder into a half stadium for increasing elongation L.

We start our numerical investigation with an array of semi-cylinders as shown in Fig. C.1a. The colored lines in Fig. C.2a show the extinction efficiency as a function of wavenumber. The systems consist of one up to five semi-cylinders in a row. By inspecting $Q_{ext}(\tilde{v})$, we observe that the sharp ripples, which can be observed in the case of a perfectly spherical or cylindrical scatterer, are not present. The black dashed line shows the approximation of Q_{ext} for a semi-cylinder given in Eq. A.19. In analogy to the 3D simulations and the measurements of the corresponding domes, we observe that the wiggle structure is not affected by the increased number of semi-cylinders. Figures C.2b-Fig. C.2f show the squared absolute value of the electric field, $I \propto |E|^2$, of the systems. The wavenumbers are selected to correspond to the peak around 3200 cm^{-1}.

Next, we evaluated the effect of two neighboring semi-cylinders (see Fig. C.1b). The result is shown in Fig. C.3. Figure C.3a shows the extinction efficiency as a function of wavenumber for two semi-cylinders with distance d of 0, 5 and 10 µm. We observe only small deviations between Q_{ext} for increasing distance between the semi-cylinders. This corresponds to what we

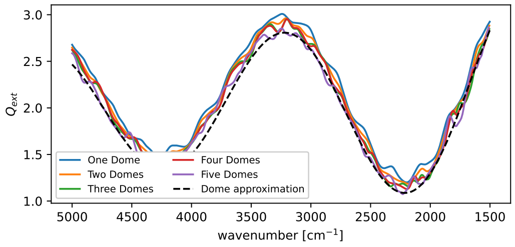

(a)

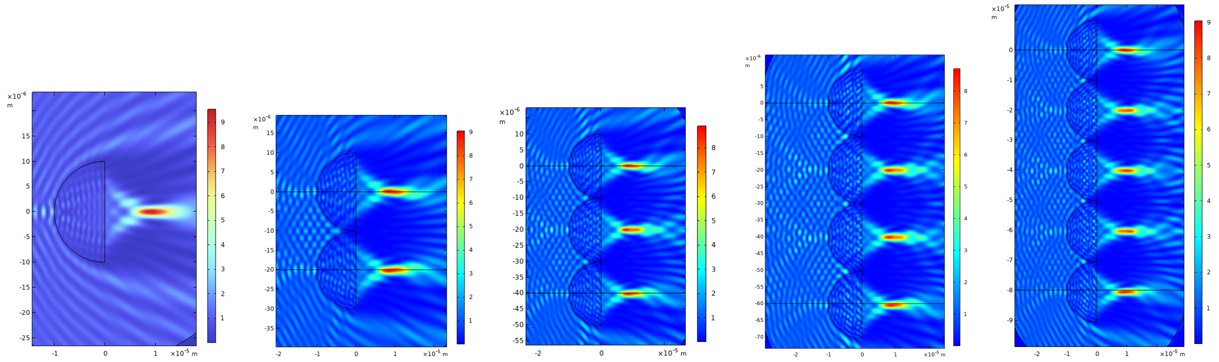

(b) $\tilde{\nu}$ = 3240 cm^{-1} **(c)** $\tilde{\nu}$ = 3200 cm^{-1} **(d)** $\tilde{\nu}$ = 3200 cm^{-1} **(e)** $\tilde{\nu}$ = 3200 cm^{-1} **(f)** $\tilde{\nu}$ = 3240 cm^{-1}

Figure C.2. The colored lines in (a) show the extinction efficiency for a system consisting of an increasing number of semi-cylinders in a row. The black line is the approximation for the extinction efficiency of a semi-cylinder, as given in Eq. A.19. The panels (b)-(f) show the intensities of the electric fields of the systems for wavenumbers that correspond to the individual system resonances in Q_{ext}, which, depending on the number of semi-cylinders in the system, occur in the vicinity of 3200 cm^{-1}.

observe for both measurements and simulations of two domes with increasing distance. The panels in Fig C.3b-Fig. C.3d show the intensity of the three situations for $\tilde{\nu}$ = 3240 cm^{-1}.

We also evaluated the system where the semi-cylinder is changed into a semi-stadium (Fig. C.1c) by increasing the elongation L from 0 to 30 µm. Figure C.4a shows the extinction efficiency for these systems. The dashed lines show the approximation for a semi-stadium. The equation for the approximation for Q_{ext} is presented in[20]. We observe that the wiggles are shifted towards shorter wavenumbers. This agrees with results presented in[20]. Measurements and simulations of a semi-capsule also show the same trend. The panels below (Fig. C.4b-Fig. C.4f), show the the intensities for wavenumbers which correspond to the resonances in Q_{ext}, located at 3240 cm^{-1} for $L = 0$ and are shifted towards lower wavenumbers as L increases.

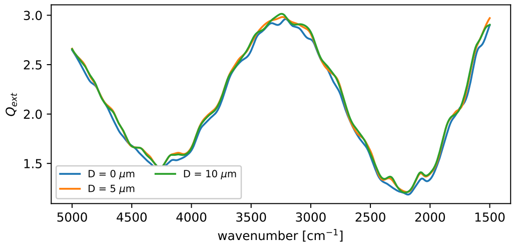

(a)

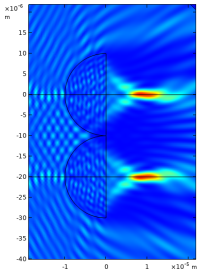

(b) $\tilde{\nu}$ = 3240 cm^{-1}, $d = 0\mu$m

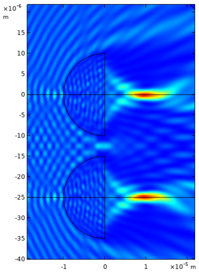

(c) $\tilde{\nu}$ = 3240 cm^{-1}, $d = 5\mu$m

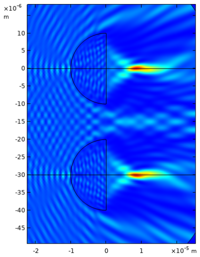

(d) $\tilde{\nu}$ = 3240 cm^{-1}, $d = 10\mu$m

Figure C.3. (a) Q_{ext} as a function of wavenumber for a system consisting of two semi-cylinders separated with a distance equal to 0 (blue line), 5 μm(orange line), and 10 μm (green line). The panels (b)-(d) show the intensities at 3240 cm^{-1}.

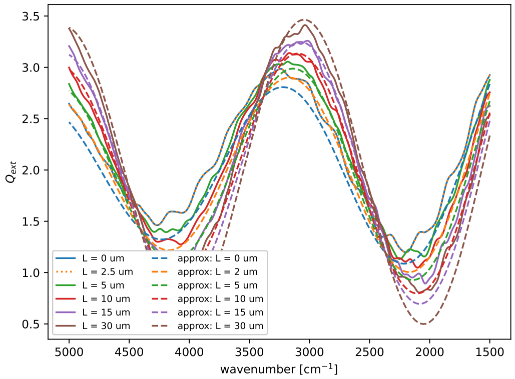

(a)

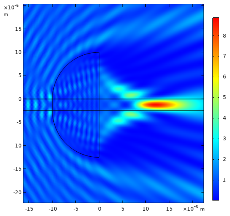

(b) $\tilde{\nu}$ = 3240 cm^{-1}, $L = 2.5\mu$m

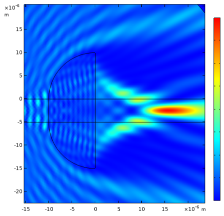

(c) $\tilde{\nu}$ = 3180 cm^{-1}, $L = 5\mu$m

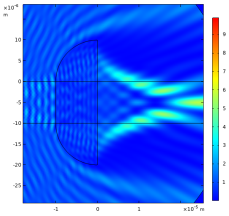

(d) $\tilde{\nu}$ = 3170 cm^{-1}, $L = 10\mu$m

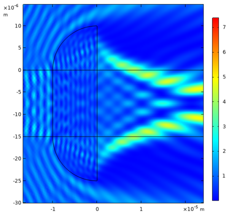

(e) $\tilde{\nu}$ = 3100 cm^{-1}, $L = 15\mu$m

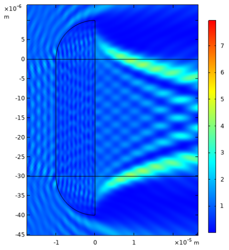

(f) $\tilde{\nu}$ = 3050 cm^{-1}, $L = 30\mu$m

Figure C.4. (a) Extinction efficiency of a semi-stadium with increasing elongation L. The dashed lines correspond to the approximation of Q_{ext} for a semi-stadium. (b)-(f) show the intensities for wavenumbers which correspond to the resonances in Q_{ext}, located at 3240 cm^{-1} for $L = 0$, and are shifted towards lower wavenumbers as L increases.
